# Conservative Therapy of External Invasive Cervical Resorption with Adhesive Systems: A 6-Year Follow-Up Case Report and Literature Review

**DOI:** 10.1155/2022/9620629

**Published:** 2022-10-28

**Authors:** Riccardo Aiuto, Gianluca Fumei, Erica Lipani, Daniele Garcovich, Mario Dioguardi, Dino Re

**Affiliations:** ^1^Department of Biomedical, Surgical and Dental Sciences, Istituto Stomatologico Italiano, University of Milan, Milan, Italy; ^2^Department of Restorative Dentistry and Endodontics, University of Insubria, Varese, Italy; ^3^Department of Dentistry, Universidad Europea de Valencia, Valencia, Spain; ^4^Department of Clinical and Experimental Medicine, University of Foggia, Foggia, Italy

## Abstract

The diagnosis and treatment of external invasive cervical resorption (EICR) could be a challenging clinical situation even for the most experienced dentists. It is a fairly rare lesion and a poorly understood phenomenon, and its insidious and aggressive nature can lead to tooth loss. Even in the era of dental implants, trying to save a compromised tooth is imperative for any clinician. This report presents a case of an upper central incisor with a class II Heithersay EICR in which treatment was performed using a multidisciplinary approach and the defect was restored with resin composite. The surgery in which the defect was exposed and repaired was followed by an endodontic treatment and the placement of a fiber-reinforced post. In this case, the use of modern materials, such as resin composites, allowed not only the avoidance of tooth extraction but also the achievement of satisfactory aesthetic results. The 6-year follow-up demonstrated the success of therapy and the resolution of clinical symptoms. This case report highlights the importance of early detection of EICR and how composite resins could provide an effective and aesthetic restauration of the defect, which favors the health of the surrounding gingival tissue.

## 1. Introduction

Root resorption is characterized by the loss of tooth hard tissues; this process can be physiological if it occurs during teething, but it is a pathological if it occurs in the permanent dentition, producing irreversible damage and/or potential tooth loss [1]. Root resorption may be classified, according to the location on the root surface, into external resorption (if it starts from the periodontal tissue) and internal resorption (if it starts from the pulp tissue). Furthermore, external root resorption can also be divided into superficial resorption, external inflammatory resorption, external invasive cervical resorption (EICR), external replacement resorption, and transient apical breakdown [2].

EICR is a clinical term used to describe a rare condition whose prevalence ranges from 0.02 to 2.3% [3] but often leads to tooth loss as a result of its insidious and asymptomatic nature [4]; the lesion may become symptomatic in advanced cases when pulp tissue is involved [5].

The lesion is characterized by a cervical location and an invasive nature. The resorptive process begins on the surface of the root below the epithelial attachment, namely, the connective tissue attachment zone [6]. Several clinical classifications were introduced to assist the clinician in treatment planning. The Heithersay classification [7] was the first to be developed, but despite its great usefulness, it relied on a two-dimensional radiograph that cannot guarantee a detailed assessment of a three-dimensional lesion. Patel et al. has recently introduced a three-dimensional classification [8]. This new classification aims to provide a more accurate estimate of the preoperative EICR condition using cone beam computed tomography (CBCT) imaging.

The etiology of EICR is complex and unclear; several factors are potentially involved, such as a history of trauma, intracoronal bleaching, orthodontic treatment, and periodontal root planning or scaling [3].

A correct diagnosis of EICR, based on a clinical and radiological examination, is an essential prerequisite for its successful treatment. Several materials have been proposed in the literature to restore the defect. In the past, in these cases, amalgam [9] and glass ionomer [7] were widely used. Currently, an approach based on biocompatible materials is preferred, such as MTA (Dentsply-Tulsa Dental, Johnson City, TN, USA) [10] or Biodentine (Septodont, Saint-Maur-des-Fossés, France) [11], and materials with high aesthetic properties, such as composite resins [12]. In the last few years, there has been a growing interest in the treatment of EICR, perhaps due to the increased use of CBCT in endodontics or also due to the aggressive and invasive nature of this type of lesion. This case report aims to illustrate the effects of a multidisciplinary approach and the use of composite resin as a restorative material in EICR.

## 2. Case Report

**Figure 1:**
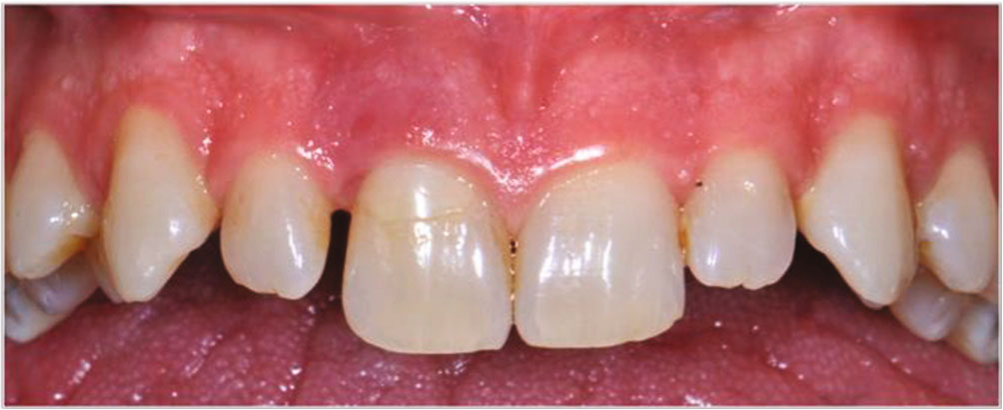
Preoperative clinical condition.

**Figure 2:**
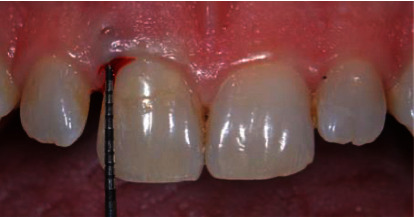
Bleeding on probing exam.

A 38-year-old male who attended the University Department of the Istituto Stomatologico Italiano (ISI) referred a slight pain during brushing of the upper left central incisor (1.1). The patient reported a good level of general health, and a detailed anamnesis did not report a history of trauma or other relevant problems. During clinical examination, a class V composite restoration was observed in the cervical third of 1.1. In the same area, the presence of bleeding on probing (Figures 1 and 2) indicated a relevant gingival inflammation. The tooth was negative to the pulp sensitivity thermal test.

**Figure 3:**
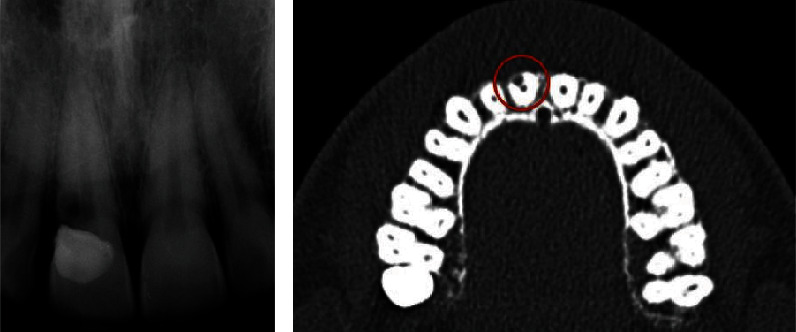
(a) Preoperative periapical X-ray and (b) preoperative CBCT.

To confirm suspicion of EICR, a radiographic examination was also realized; first of all, a periapical radiograph was performed and showed the presence of a radiolucent lesion in the coronal third of the root of 1.1, in its distal portion (Figure 3(a)). However, in this case, CBCT imaging was essential to assess the stage of the lesion and which structures were compromised (Figure 3(b)). 3D images revealed that the lesion had already penetrated close to the coronal pulp, but it appears that there was no communication. Considering the clinical and radiological findings, the resorptive defect was classified as a Heithersay class II.

Due to the importance of saving a natural tooth and the aesthetic value of a central incisor, all treatment options were discussed with the patient, and finally, the external approach was chosen. That included a surgical phase, endodontic treatment, and finally replacement of the previous restoration. The informed consent form was obtained from the patient prior to the beginning of treatment.

The main phases of the clinical procedure are reported as follows.

### 2.1. First Session: Surgical Step

**Figure 4:**
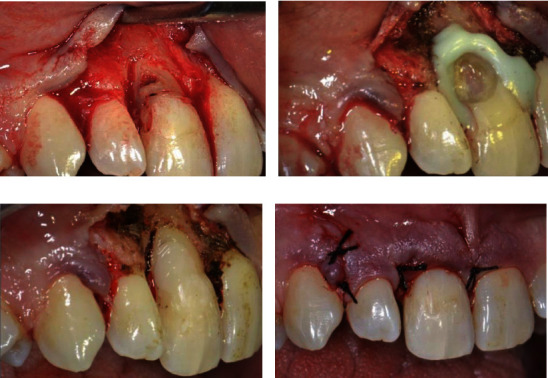
Phases of surgery: (a) defect exposition by surgical flap, (b) isolation by liquid rubber dam, (c) composite restoration of the defect, and (d) repositioning and suture of the flap.

The complexity of this pathology requires the use of an operating microscope and the exposition of the defect by a surgical flap. After administering local anesthesia (articaine 4% with epinephrine 1 : 100,000, Pierrel, Capua, Italy), a full thickness flap was performed (Figure 4(a)). Once the defect was exposed, the resorptive granulation tissue was accurately removed, and the site was debrided and cleaned with surgical straight handpieces (Ultimate Power+, B.A. International, Fiumana, Italy) and with a round tungsten carbide ball bur. Once hemostasis was managed, isolation with a liquid rubber dam was obtained (Figure 4(b))), and common steps were followed for composite restoration.

First, a dentin etching was performed for 15 seconds with 37% phosphoric acid, and a two-step bonding system (Scotchbond Universal Adhesive, 3M Company, St. Paul, MN, USA) was applied and light cured for 40 seconds. Subsequently, the defect was filled and restored with flowable composite (Filtek™ Universal Restorative, 3M Company, St. Paul, MN, USA), and the restoration was accurately finished and polished (Figure 4(c))). Finally, the flap was repositioned with interrupted 4\0 silk suture (Perma-Hand™, Ethicon Inc., Johnson & Johnson Company, Somerville, NJ, USA; Figure 4(d)). Ice application was recommended, and a twice-daily oral rinse with 0.20% chlorhexidine was prescribed during the 7 days after surgery.

### 2.2. Second Session: Endodontic Step

Due to the negative pulp response, endodontic treatment was required. In this appointment, soft tissue healing was checked, sutures were removed, and root canal therapy was performed.

**Figure 5:**
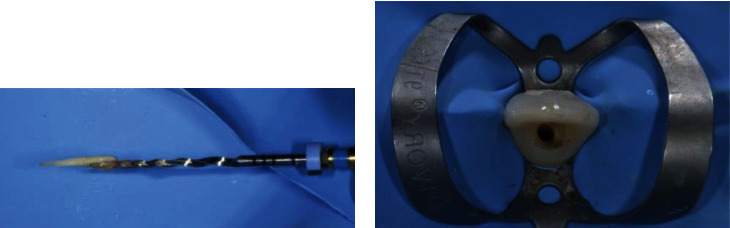
(a) Necrotic pulp tissue and (b) rubber dam isolation.

First, local anesthesia (articaine 4% with epinephrine 1 : 100,000, Pierrel) was administered, and a rubber dam (Nic Tone, Manufacturera Dental Continental S.A. De C.V., Zapopan, Mexico) isolation was placed (Figure 5(b)) with a 9 clamp (Ivory®, Kulzer Nordic AB, Helsingborg, Sweden) fixed on tooth 1.1. The cleaning and shaping of the root canal were carried out with a rotary file (ProTaper Gold, Dentsply Maillefer, Ballaigues, Switzerland), 5% NaOCl irrigation (NICLOR 5, OGNA, Muggiò, Milan, Italy), and 17% EDTA (OGNA, Muggiò).

**Figure 6:**
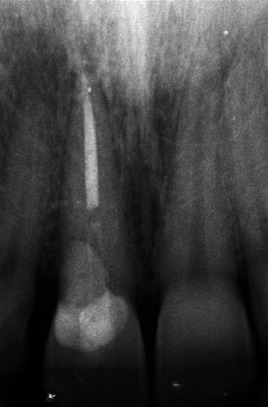
Postoperative X-ray of root canal treatment.

In Figure 5(a), it is possible to appreciate the characteristic grey color of a necrotic pulp tissue. After drying the canal with paper points, the root canal was filled with a 40# ProTaper Gold master cone and cement (AH Plus^®^, Dentsply Sirona, Lancaster, A, USA) using the continuous wave of condensation technique. Once the canal was definitely filled, the post space was realized, and a fiber-reinforced post was subsequently inserted. Postoperative X-ray was performed (Figure 6).

### 2.3. Third Session: Restorative Step

**Figure 7:**
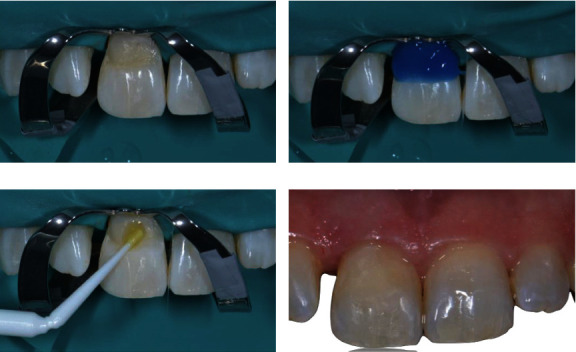
Phases of class V restoration: (a) rubber dam isolation, (b) enamel total acid etching, (c) bonding agent application, and (d) final restoration results.

The patient was recalled after 3 months. On clinical examination, the periodontal probing revealed healthy periodontal tissues, without bleeding and a probing depth of less than 3 mm. The class V restoration was replaced, on the patient's request. Rubber dam isolation was placed with No. 9 clamp on tooth 1.1 and No. 0 clamp on tooth 2.5 (Figure 7(a)).

The old resin restoration was removed, and total acid etching was performed for 20 seconds (Figure 7(b)). Then, a two-step bonding system (Scotchbond Universal Adhesive, 3M Company) was applied to the cavity (Figure 7(c)). The cavity was filled with resin composite (Filtek Z500, 3M Company), and finally, the restoration was polished and finished (Figure 7(d)).

**Figure 8:**
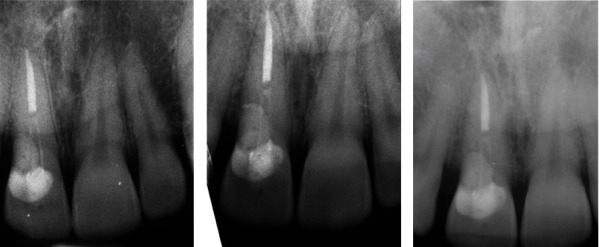
X-ray at (a) 1-year, (b) 2-year, and (c) 3-year follow-ups.

Subsequently, the patient received monitoring visits during which clinical examination, and radiographs were performed; follow-up has been carried out, respectively, after 1, 2, and 3 year, and at each appointment, a periapical radiograph was taken (Figure 8).

**Figure 9:**
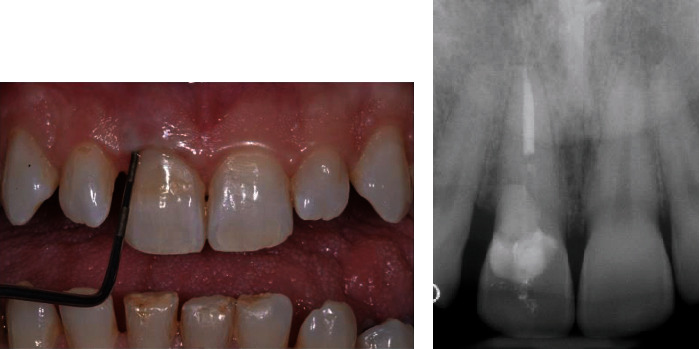
(a) Probing and postoperative clinical condition and (b) X-ray at the 6-year follow-up.

Six years later, the patient was checked again, and the 6-year follow-up confirmed the treatment success; indeed, as is apparent in Figure 9, it showed that the absence of sign of gingival inflammation, no bleeding on probing, the absence of periodontal pocket, complete remission of symptoms, and the X-ray examination indicated that no bone loss occurred.

## 3. Discussion

Diagnosis is a key step when facing an insidious lesion, such as EICR; clinician may initially be disorientated, since a radiolucent image on the X-ray could be misdiagnosed as a subgingival caries. However, there are some distinctive features that allow the clinician to establish a differential diagnosis: a carious lesion in a healthy periodontium does not usually present bleeding on probing, which is usually observed instead in the case of EICR due to the high vascularization of the granulation tissue [6]; moreover, there is a typical pinkish discoloration (pathognomonic clinical sign) of the crown in the cervical region of the affected tooth, and in addition, the resorbed area produces a scraping sound on probing giving the feeling of hard tissue, quite different from the one that characterizes the softened dentine [7].

The introduction of CBCT imaging in dentistry, especially in endodontics, plays an important role in the diagnosis and management of pathologies, such as EICR; however, considering that the patient's radiation exposure should be kept “as low as reasonably achievable,” the use of CBCT imaging should be limited to the cases in which the information gathered by conventional imaging systems is not enough.

At present, CBCT imaging has been recommended for the planning of endodontic surgery treatment [13–15]; indeed only with a 3D imaging, the clinician can assess the exact location and extension of the lesion, and therefore its real restorability. Furthermore, the increased precision of the CBCT imaging can also result in early detection of EICR and, in these cases, is of great importance [16, 17].

As previously reported, in the present case report, the treatment of a Heithersay class II lesion was performed. The Heithersay classification was the first developed, in which EICR lesions were classified into four classes according to the size and extension of the defect into dentine: from a small resorptive lesion in the cervical area (Class I) to an extensive resorptive defect beyond the coronal third of the root (Class IV) [3].

Depending on the severity and type of injury, the treatment plan will radically change from an external approach to an internal approach, up to orthodontic extrusion or, in some cases, even tooth extraction. Today, there are no clear treatment guidelines for EICR, and for this reason, clinical classifications, such as the Heithersay class I, are so useful. However, this classification was developed on two-dimensional images, which could offer an underestimated representation of the real situation. To overcome this limitation, Patel et al. introduced a three-dimensional classification based on CBCT findings [8]. Three parameters were considered in this new classification: lesion height (1: at CEJ level or coronal to the bone crest (supracrestal), 2: extends into the coronal third of the root and apical to the bone crest (subcrestal), 3: extends into the middle third of the root, and 4: extends into the apical third of the root), circumferential spread (*A*: ≤90°, *B*: ≤180°, *C*: ≤270°, and *D*: >270°), and proximity to the root canal (d: lesion confined to dentine and p: probable pulpal involvement) [8]. Following these parameters, it is possible to classify EICR defects in three dimensions, thus improving communication between colleagues, prognostic, and treatment outcomes.

A careful removal of the resorptive tissue and thorough root canal cleaning [18] are absolutely necessary to guarantee a successful treatment outcome; regarding the restoration of the defect, as previously discussed, several materials have been proposed. Several studies have been conducted in recent years on the qualities and clinical performance of mineral trioxide aggregate “MTA” (Dentsply-Tulsa Dental); many favorable properties were described, among the most important being biocompatibility and optimal tissue response [10, 19].

Although several articles reported that Biodentine (Septodont), a calcium silicate-based product, has better physical properties (resistance and compressive strength) and handling properties than MTA, in fact, Biodentine was developed from MTA technology in order to improve some characteristics of these types of materials [11, 20].

Even if the efficacy of Biodentine and MTA is widely demonstrated, in this case report, composite resin (Filtek™ Universal Restorative, 3M Company) was preferred. Both the “white” and “pink” aesthetics of a smile are complex to imitate, especially if there is an impairment of the dental and periodontal tissues that entail the risk of losing a tooth in the aesthetic area [21]. However, modern composite resins offer a very satisfactory degree of integration when it is possible to restore the natural tooth. Adequate adhesive bond strength is ensured in root dentine by employing a proper self-etch adhesive system [12].

Additionally, composite resin has a faster setting time (20 seconds light-curing time) compared to the long setting time of the MTA. Moreover, the polishing of the restoration provides a smooth surface that prevents the accumulation of subgingival plaque, unlike the rough surface MTA. Although composite resins are not fully biologically acceptable for periodontal tissues, the “creeping attachment” described by Goldman and Cohen [22] develops, and coronal migration of gingival marginal tissue occurs [23]. In this case, the absence of gingival inflammation and the complete resolution of the symptoms support the clinical assessment of a new and healthy epithelial tissue gingival attachment.

## 4. Conclusions

Early detection of EICR lesion and an accurate endodontic and restorative treatment planning are paramount to achieve a favorable clinical outcome. This case report shows how accurate removal of resorption tissue followed by defect restoration with composite resins could be a good choice for EICR treatment in aesthetic zones. As reported, this treatment approach could result in a favorable periodontal tissue response. More research is needed to confirm the long-term outcome of this treatment modality.
